# Key points in preventing tobacco use among adolescents

**DOI:** 10.1186/1617-9625-8-1

**Published:** 2010-01-05

**Authors:** Constantine Vardavas

**Affiliations:** 1 Associate Editor, Prevention and Clinical Sciences

## 

Tobacco use is the largest global threat to public health and is anticipated to kill 1000 million people prematurely this century [1]. Despite this grim fact, millions of lives can be saved if urgent action is taken towards preventing cigarette experimentation and subsequent nicotine addiction among adolescents. In the European Union alone, 19.8% of 13-15 year olds are current tobacco users, while one in three non-smokers of the same age, report susceptibility to smoke within the next year [2]. This exact youth experimentation is what the tobacco industry's future prosperity depends on, a fact that the multinational tobacco industry has acknowledged [3].

Substantial research by health professionals, and public health policy advocates have indicated different avenues through which adolescent smoking can be prevented at a population based level, key points which are depicted in the Tobacco Control Funnel in Figure 1.

**Figure 1:**
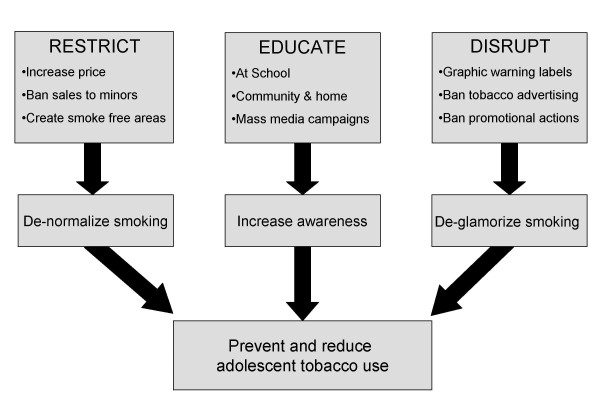
**Tobacco Control Funnel**.

## Restrict

One of the most discussed ways of reducing tobacco use among adolescents is by increasing the price of tobacco products, through a regulated rise in taxation [4]. Adolescents are very price sensitive, with the price of the cigarette pack shown to significantly influence the decision to start and quit smoking [5-8]. As a significant share of pocket money among teen age smokers is allocated towards tobacco products a price increase also reduces also the spending capacity of the adolescent. It must be stated though that price increases can be rather unpredictable as youth smoking behaviors are not as intense or consistent as adult smoking, while social sources are also a common way that adolescents obtain cigarettes.

Restricting smoking areas also play a key role in promoting the populations health, not only by reducing the negative ramifications of exposure to second hand smoke but also by reducing cigarette consumption by restricting the smoker's ability to smoke under certain situations. Furthermore, smoke free legislations are also associated with a increased rate of smoking cessation [7]. This environment that prohibits smoking has an important impact on the perception of the acceptance of smoking at a population based level by reducing the visibility of role models who smoke [8]. Smoking bans in other areas, such as the household or schools also have this desired effect on adolescent smoking experimentation, a fact which should be acknowledged [9].

## Disrupt

Industry advertising and brand imagery are also associated with smoking susceptibility among adolescents, with the implementation of advertising bans a core element of comprehensive tobacco control measures and a central part of the Framework Convention on Tobacco Control (FCTC) [10].

Research has indicated that adolescent smoking rates are higher in areas with a higher density of tobacco advertisements, thus increasing the community and social acceptance of smoking as part of daily life [11]. In addition to such outdoor and point of purchase advertising, the cigarette package is a critical communication device for creating and reinforcing brand imagery. Using striking colors, distinctive fonts and carefully crafted materials it is the link between other forms of tobacco advertising and the uptake of the addictive drug nicotine from a cigarette [12]. Tobacco warning labels counterattack this effect. Specifically, graphic warning labels are unique among tobacco control initiatives implemented to educate and prevent smoking initiation, as they cost little to produce and can be integrated with larger educational interventions such as mass media campaigns. Indeed research based on the theory of planned behavior among adolescents has indicated that graphic warning labels are regarded by adolescents as more effective in preventing them from smoking, and in informing them about the health effects of smoking, in comparison to text-only warnings [13]. Their effectiveness is warranted by the tobacco industry's possible actions so as to circulate disproportionately fewer cigarette packs with graphic warnings judged as more disturbing as recently published in *Tobacco Induced Diseases *by Wilson N et al., who indicated an abnormal distribution of such graphic warning labels within a Canadian community [14].

## Educate

The third aspect of the Tobacco Control Funnel for preventing adolescent smoking is to educate the adolescent, the parent and the community. School based educational programmes that focus on health promotion and tobacco use, are a key element of this avenue and their importance in health education has been stressed by previous researchers [15]. As close friends, older siblings and parents are also strong determinants of smoking during adolescents, [16] such educational programmes should not be limited to school children but through the implementation of community and mass media campaigns reach all members of society regardless of age and educational status. Furthermore such mass media campaigns can incorporate issues such as the negative short and long term health aspects of tobacco use, tobacco industry manipulation, changes in legislative actions and thus act as the connecting device between public health advocacy and the public itself.

Taking all the above into account, the need for collecting translational research at a national level is imperative. This community oriented research is needed to provide the society and public health advocates with the original national data required so as to de-normalise tobacco use and prevent adolescent smoking on a country to country basis, an action which *Tobacco Induced Diseases *strongly supports.
